# Palmitate-induced insulin resistance is attenuated by Pioglitazone and EGCG through reducing the gluconeogenic key enzymes expression in HepG2 cells

**Published:** 2017

**Authors:** S Yadollah, N Kazemipour, S Bakhtiyari, S Nazifi

**Affiliations:** * Department of Basic Science, School of Veterinary Medicine, Shiraz University, Shiraz, Iran; ** Department of Clinical Biochemistry, Faculty of Medicine, Ilam University of Medical Sciences, Ilam, Iran; *** Department of Clinical Pathology, School of Veterinary Medicine, Shiraz University, Shiraz, Iran

**Keywords:** gluconeogenesis, epigallocatechin gallate (EGCG), pioglitazone, phosphoenolpyruvate carboxykinase, glucose 6-phosphatase

## Abstract

Hypothesis: Palmitate causes insulin resistance (IR) in insulin target tissue. Pioglitazone (an anti-hyperglycemic agent) and epigallocatechin gallate (EGCG, a dietary supplement) can be used for the treatment of type 2 diabetes. However, their molecular effects on gluconeogenesis remain unclear.

Objective: Hence, we aimed to investigate the simultaneous effect of these anti-hyperglycemic agents on gluconeogenesis through in vitro experiments.

Methods: HepG2 cells were treated with 0.5 mM palmitate, 10 μM pioglitazone, and 40 μM epigallocatechin gallate (EGCG). Gene expression assay was used to investigate the underlying mechanism. Glucose production assay was applied in culture medium to evaluate the activity of gluconeogenesis pathway.

Results: Palmitate induced IR could significantly increase G6Pase and PEPCK gene expressions by 58 and 30%, respectively, compared to the control. EGCG reduced the expression of PEPCK and G6Pase by 53 and 67%, respectively. Pioglitazone reduced the mRNA level of PEPCK and G6Pase by 58 and 62% respectively. Combined treatment of insulin-resistant cells with EGCG and pioglitazone significantly decreased the mRNA level of PEPCK and G6Pase by 73 and 80%, respectively. Treatment with palmitate increased glucose production by 50% in HepG2 cells. When the insulin resistant HepG2 cells were treated alone with EGCG and pioglitazone, the glucose production reduced by 50 and 55%, respectively. The combined treatment with EGCG and pioglitazone resulted in 69% reduction in glucose production compared to the palmitate treated HepG2 cells.

Conclusions: These data suggest the additive inhibitory effect of co-treatment with pioglitazone and EGCG on the gluconeogenesis pathway in palmitate-induced insulin resistance HepG2 cells.

## Introduction

Nowadays, calorie rich dietary habits and sedentary lifestyle are possible common predisposing factors resulting in diabetes [**1**]. Insulin resistance is a central feature of type 2 diabetes, which results from reduced responsiveness of insulin target tissues such as liver, adipose and muscle to normal insulin levels [**2**][**3**]. Increased level of free fatty acids (FFAs) has been proposed as an important etiology of insulin resistance [**2**][**4**]-[**7**]. It has been shown that lipid oversupply can induce insulin resistance in muscle and liver, which is mediated by increased level of FFAs [**2**][**8**][**9**]. The activity of gluconeogenesis pathway is increased in insulin resistance and type 2 diabetes resulting in increased fasting hepatic glucose production [**10**]. Given the destructive role of increased gluconeogenesis in diabetic patients, phosphoenolpyruvate carboxykinase (PEPCK) and glucose 6-phosphatase (G6Pase), two key enzymes in gluconeogenesis, would most likely be affected [**4**][**6**]. Unfortunately, until now, the synthetic drugs could not completely overcome diabetes complications [**11**].

In the last decades, a large number of studies focused on introducing herbal drugs for the treatment of diabetes, but the simultaneous effect of herbal and synthetic drugs on the hepatic cells could be given outstanding results [**12**]. It has been reported that the green tea is one of the important herbal agents that can lower the blood sugar [**5**]. Epigallocatechin gallate (EGCG) is the most important fractions of green tea, which has been shown to decrease the blood sugar. Thiazolidinediones (TZDs), the peroxisome proliferator-activated receptor γ (PPARγ) agonists, are one of the insulin-sensitive drugs that have been used for the treatment of type 2 diabetes [**4**][**13**]. At the molecular level, TZDs activate PPARγ, a ligand-activated nuclear transcription factor that modulates the expression of a number of genes encoding proteins involved in glucose and lipid metabolism. PPARγ receptors are most strongly expressed in adipose tissue and the vascular wall, with secondary benefits on insulin sensitivity in skeletal muscle and liver. It has been reported that pioglitazone enhances glucose uptake by muscle and adipose tissue, and to a lesser degree, reduces hepatic gluconeogenesis. It also promotes adipogenesis, resulting in an increased uptake of FFAs and glucose, and a decreased release of FFAs into the circulation [**14**], leading to reduced hepatic and muscular FFA flux and further reduction of gluconeogenesis, and increased glucose uptake. More recent evidence also suggests that pioglitazone improves short-and long-term pancreatic beta cell function [**8**], thus reducing the functional stress associated with chronic hyperinsulinemia in type 2 diabetes. However, the simultaneous effects of EGCG and pioglitazone, in hepatic cell gluconeogenesis, have not been studied yet, and this study aimed to investigate the simultaneous effects of EGCG and pioglitazone on gluconeogenesis pathway in palmitate-induced insulin resistant HepG2 cells.

## Methods

**Cell culture**

HepG2 cells were purchased from The Pasteur Institute of Iran and were cultured in low-glucose Dulbecco’s modiﬁed Eagle’s medium (DMEM) (Gibco, Berlin, Germany) supplemented with 10% FBS,2 mM glutamine, 100 units/mL penicillin, 100 μg/mL streptomycin and maintained under 5% CO2 and 37°C.

**Palmitate treatment**

Insulin resistance was induced by palmitate treatment as described in previous studies [**2**][**15**]-[**20**]. In brief, sodium palmitate was dissolved in prewarmed 50% (v/v) ethanol, then diluted in prewarmed DMEM containing 1% (w/v) fatty acid-free BSA to ﬁnal concentration and placed in an incubator for 2 hr with shaking. The same concentration of ethanol mixed BSA (1%) was administrated to control cells. EGCG and pioglitazone were added 2–3 hr before the incubation of the cells with palmitate.

**Gene expression analysis**

Total RNA was extracted using RNeasy mini kit. Total RNA (1 μg) was reverse transcribed using Fermentase reverse transcriptase. Real-time PCR was conducted using a RotorGene Q instrument (Corbett Research, Australia). The complementary DNA was ampliﬁed in duplicate using QuantiTect primers and QuantiFast SYBR Green PCR Master Mix. The data were normalized against β-actin transcript level and analyzed by delta-delta Ct method. The primer sequences of target genes used in this study are listed in **Table 1**.

**Table 1:** Primer sequence of target genes

	Forward 5 to 3	Reverse 5 to 3
PEPCK	TGACAACTGCTGGTTGGCT	TGGTGCGACCTTTCATGC
G6Pase	GGGAAAGATAAAGCCGACCTAC	CAGCAAGGTAGATTCGTGACAG
β-actin	TTCTACAATGAGCTGCGTGTG	GGGGTGTTGAAGGTCTCAAA

**Glucose production assay**

Glucose production assay was used to evaluate the activity of gluconeogenesis pathway [**21**]. Brieﬂy, cells were seeded in 24-well plates and washed three times with PBS to remove glucose, incubated with 1 nM insulin for 16 hr in 300 μl of glucose production medium (glucose- and phenol red-free DMEM containing gluconeogenic substrates, 20 mM sodium lactate, and 2 mM sodium pyruvate). A quantity of 250μl of the medium was sampled for the measurement of glucose concentration using a glucose assay kit (Sigma–Aldrich). Glucose concentration was normalized with the total protein content determined from the whole cell lysates.

**Statistical Analysis**

The data are presented as mean ± SD of at least three independent experiments. The statistical analyses were applied using SPSS 19.0 (SPSS, Chicago, IL). Comparisons between all groups were performed with one-way analysis of variance (ANOVA) test. If signiﬁcant differences were found, Tukey’s post hoc test was applied. Values of p<0.05 were considered statistically signiﬁcant.

## Results

**EGCG and pioglitazone treatment reduced gluconeogenesis activity in insulin-resistant HepG2 cells**

**Fig. 1:**
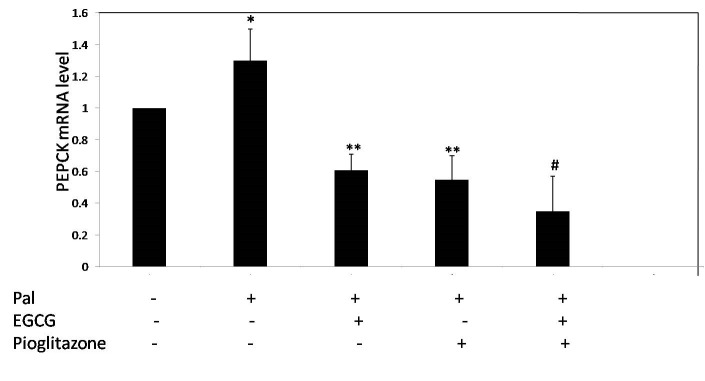
The effect of EGCG and pioglitazone on mRNA level of PEPCK. HepG2 cells were incubated for 24 h with palmitate (0.5 mM), 10 μM pioglitazone and 40 μM EGCG. Total RNA extracted, and then, the level of PEPCK expression was determined by real-time PCR and normalized to endogenous β-actin. *p < 0.05 versus untreated cells, **p < 0.05 versus just palmitate-treated cells, ≠<0.05 Combined treated cells versus the cells individually treated with palmitate and EGCG or Pioglitazone. The ﬁgure shows representative data gained from mean ± SD of three independent experiments.

**Fig. 2:**
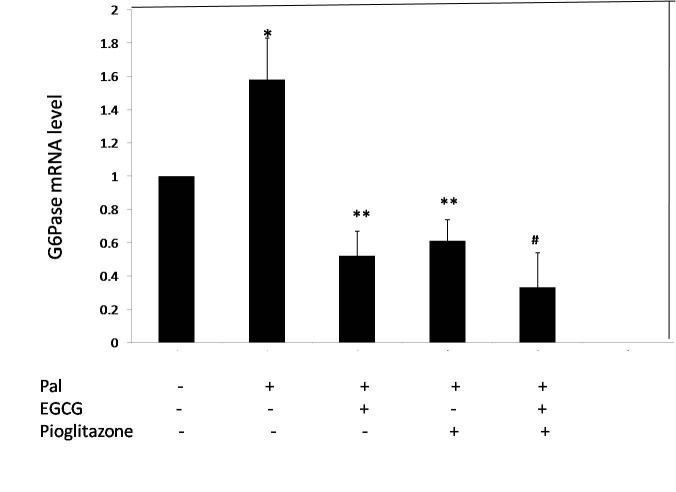
The effect of EGCG and pioglitazone on mRNA level of G6Pase. HepG2 cells were incubated for 24 h with palmitate (0.5 mM), 10 μM pioglitazone and 40 μM EGCG. Total RNA extracted, and then, the level of G6Pase expression was determined by real-time PCR and normalized to endogenous β-actin. *p < 0.05 versus untreated cells, **p < 0.05 versus just palmitate-treated cells, ≠<0.05 Combined treated cells versus the cells individually treated with palmitate and EGCG or Pioglitazone. The ﬁgure shows representative data gained from mean ± SD of three independent experiments.

The mRNA level of G6Pase and PEPCK genes were measured to evaluate the effect of EGCG and pioglitazone on gluconeogenesis. First, the cells were incubated in the presence of 0.5 mM palmitate to induce insulin resistance. As shown in **Fig. 1**, palmitate increased mRNA level of G6Pase and PEPKC by 58 and 30% respectively compared to control. EGCG 40 μM reduced the expression of PEPCK and G6Pase by 53 and 67%, respectively (**Fig. 1** and **Fig. 2**). As shown in **Fig. 1** and **2**, 10 μM pioglitazone reduced the mRNA level of PEPCK and G6Pase by 58 and 62%, respectively. Combined treatment of insulin-resistant cells with EGCG and pioglitazone significantly reduced the mRNA level of PEPCK and G6Pase by 73 and 80%, respectively (**Fig. 1** and **Fig. 2**).

**EGCG and Pioglitazone significantly reduced glucose production in HepG2 cells under insulin resistance condition**

**Fig. 3:**
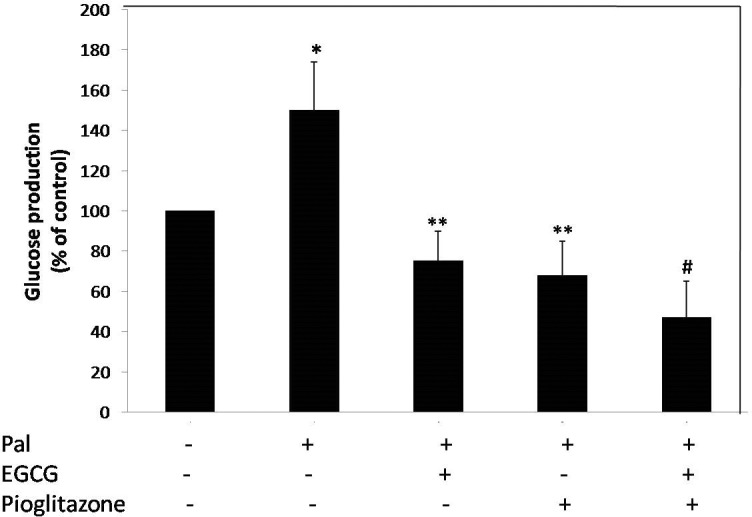
The effect of EGCG and pioglitazone on glucose production in HepG2 cells. The cells were incubated for 24 h with palmitate (0.5 mM), 10 μM pioglitazone and 40 μM EGCG in serum-free DMEM. After incubation for 24 h, the medium was replaced with glucose-free DMEM, and then the cells were incubated with 1 nM insulin for 16 h. The glucose level was determined using a glucose assay kit as described in the Materials and methods section. *p < 0.05 versus untreated cells, **p < 0.05 versus just palmitate-treated cells, ≠<0.05 Combined treated cells versus the cells individually treated with palmitate and EGCG or Pioglitazone. The ﬁgure shows representative data gained from mean ± SD of three independent experiments.

As shown in **Fig. 3**, the treatment of the cells with 0.5 mM palmitate increased glucose production by 50% of the control. When the cells were pretreated alone with EGCG and pioglitazone, the glucose production reduced by 50 and 55%, respectively. The combined treatment with EGCG and pioglitazone resulted in 69% reduction in glucose production compared to control HepG2 cells (**Fig. 3**).

## Discussions

Insulin resistance is the most important feature of T2DM, which is characterized by reducing sensitivity or responsiveness of muscle, liver, and adipose tissue to the metabolic actions of insulin [**22**][**23**]. Increased plasma levels of FFA in obesity and T2DM has been shown to be correlated with insulin resistance [**15**]-[**17**][**24**][**25**]. Elevated hepatic glucose production is one of the pathophysiological consequences of insulin resistance in the liver, which is the inability of insulin to inhibit hepatic glucose production or gluconeogenesis [**4**][**7**][**22**][**26**][**28**]. Different approaches have been suggested to attenuate the insulin resistance-induced hepatic glucose production, including synthetic and herbal drugs. In the present study, we aimed to examine the simultaneous effect of EGCG and pioglitazone on gluconeogenesis activity in HepG2 cells under insulin resistance condition. We first showed that palmitate causes insulin resistance in HepG2 cells. This finding is in agreement with the previous study [**2**]. Then we investigated the role of insulin resistance on key regulators of gluconeogenesis pathway, G6Pase and PEPCK [**29**]. IR significantly increased the expression of both enzymes compared to control. This finding was in line with the previous study [**30**][**31**].

In the next step, we determined the effect of EGCG on glucose production and expression level of G6Pase and PEPCK and in HepG2 cells under IR condition. EGCG is the main catechin of green tea that has an inhibitory effect on gluconeogenesis, and previous studies have shown a negative effect of EGCG on gluconeogenesis pathway [**32**]. Waltner-Law and colleagues reported that EGCG could repress hepatic glucose production in H4IIE rat hepatoma cells [**33**]. Collins et al. also reported that lower concentrations of EGCG could suppress hepatic gluconeogenesis through an independent insulin-signaling pathway by stimulation of 5' AMP-activated protein kinase (AMPK) [**32**]. It has also been reported that EGCG can increase insulin secretion and sensitivity in β and liver cells respectively [**34**][**35**] and inhibit hepatic gluconeogenesis in an insulin-independent manner [**36**]. Li et al. showed that EGCG ameliorated FFAs-induced peripheral insulin resistance in vivo, and this might be through decreasing oxidative stress and protein kinase Cθ (PKCθ) membrane translocation, activating the AMPK pathway and improving insulin-signaling pathway in vivo [**22**]. Cordero-Herrera et al. showed that Cocoa and epicatechin decreased PEPCK gene expression in HepG2 cells. They found that Cocoa and epicatechin had insulin-like effects and decreased glucose production and suppressed PEPKC through protein kinase B (PKB, also known as AKT) and AMPK pathways [**37**]. Similar findings were also reported for EGCG both in vitro and in vivo [**38**][**39**]. In agreement with these studies, we also found that EGCG down-regulates the expression of PEPCK and G6Pase and reduces glucose production under IR condition in HepG2 cells. Previous studies have shown that thiazolidinediones activate PPARs that are extensively expressed in adipose tissue, vascular endothelium, muscles, and liver and sensitize liver and muscle to insulin action [**40**]. Thus, we evaluated the effect of pioglitazone on gluconeogenesis pathway and glucose production in HepG2 cells under IR condition. In 2004, Scheen et al. reported that pioglitazone is an oral antihyperglycemic drug for the treatment of type 2 diabetes mellitus [**41**]. Pioglitazone was also shown to improve the long and short-term beta cell function and increase the sensitivity of liver, adipose tissue, and muscular system to insulin [**8**][**14**][**42**]. It has been reported that PPARγ stimulation results in increased glucose consumption and insulin sensitivity and decreased glucose production [**10**]. It has also been reported that pioglitazone improved glucose absorption by muscular and adipose tissues and decreased hepatic gluconeogenesis through downregulation of PEPKC and G6Pase [**43**]. An animal study reported that troglitazone resulted in a dose-dependent reduction in glucagon-stimulated gluconeogenesis in the absence of insulin. In addition, combined treatment of troglitazone with insulin produced an additive inhibition of gluconeogenesis during glucagon-stimulated conditions [**38**]. These findings are in line with ours, in which thiazolidinedione decreased gene expressions. As reported by these studies, we also found that pioglitazone down-regulated the expression of PEPCK and G6Pase and glucose production in HepG2 cells under insulin-resistance condition. In addition to these findings, we also evaluated the combined effect of pioglitazone and EGCG on gluconeogenesis pathway in HepG2 cells under insulin resistant condition. It has been reported that combined treatment of pioglitazone with metformin or sulfonylurea resulted in significant reduction of HbA1c and blood glucose concentration [**44**][**45**]. For the first time, we found in this study that the combined treatment of HepG2 cells with pioglitazone and EGCG results in significant reduction of PEPCK and G6Pase and glucose production compared to individual treatment in HepG2 cells under insulin resistance condition. We also found that the effect of EGCG is somehow identical to pioglitazone.

Overall, these ﬁndings extend the potential utility of combined treatment of HepG2 cells with pioglitazone and EGCG for the treatment of diabetes complications. However, further in vivo and clinical trial studies are required to demonstrate whether this combination therapy can efficiently prevent gluconeogenic pathway and hepatic glucose production under insulin resistance condition.

## Conflict of interest

The authors declare no conflict of interest.
